# Construction of a high-density genetic map and fine mapping of a candidate gene locus for a novel branched-spike mutant in barley

**DOI:** 10.1371/journal.pone.0227617

**Published:** 2020-01-08

**Authors:** Weibin Wang, Junyu He, Shengwei Chen, Peng Peng, Wei Zhong, Xintian Wang, Tingting Zhang, Yuping Li

**Affiliations:** College of Agronomy and Biotechnology, Yunnan Agricultural University, Kunming, Yunnan, China; USDA, UNITED STATES

## Abstract

A Yunnan branched-spike (Ynbs) barley mutant is useful for study of the genetic mechanisms underlying variation in barley spike architecture. In the current study, a mutant (Ynbs-1), a recombinant inbred line (RIL-1), and a cultivar (BDM-8) were used as parents to develop populations. Ynbs-1 exhibits typical branched spike, whereas the others exhibit six-row spike. Genetic analysis on their F_1_, F_2_ and F_3_ populations showed that one recessive gene is responsible for the branched spike trait. SLAF marker generated from specific locus amplified fragment sequencing (SLAF-seq) was used to genotype the populations. A high-density genetic map of barley was constructed using 14,348 SLAF markers, which covered all 7 chromosomes at 1,347.44 cM in length with an average marker density of 0.09 cM between adjacent markers. Linkage analysis of the branched-spike trait using the genetic map indicated that branched spike trait in the Ynbs-1 is controlled by single locus on chromosome 2H at the interval between 65.00 and 65.47 cM that is flanked by Marker310119 and Marker2679451. Several candidate genes that may be responsible for barley multiple-spikelet degeneration, single-floret spikelet increase and seed set rate decrease were identified in the region. The high-density genetic map and the gene locus revealed in this study provide valuable information for elucidating the genetic mechanism of spike branching in barley.

## Introduction

During its long evolutionary process, barley has established a typical non-branching spike structure with only a triple spikelet on each rachis node of a spike [1]. However, the typical barley spike structure can be effectively remodeled by gene mutation due to natural factor [2–3], exogenous DNA [4], physical mutagen [5] or chemical mutagen [6], resulting in branched spikes with stable heredity. In barley mutants, branched spike can replace the triple-spikelet that grow on the main rachis, and multiple spikelet can grow only on the rachis of the branched spike [2–4,6]. By observing the developmental process of young spike, the branched-spike primordia could effectively replace the triple-spikelet primordia, differentiate into branched spike, and then generate multiple spikelet on the rachis of the branched spike in Poly row branched spike (Prbs) [7] and Ynbs mutant [8]. Because the normal path of young spikelet development can be changed and the growth space of multiple spikelet can be expanded effectively, branched-spike mutant is considered important genetic resource for studies of the genetic mechanisms underlying barley spike architectural variants [6,9].

After Larsson [2] reported the Foma mutant of barley, which has branched spikes, three branched-spike mutants, F_151_ [3], Prbs [4] and Compositum [10] were also reported successively. In most of the mutants, branched spike was controlled by single recessive gene [2,10–11]. In the Foma mutant, the branched spike was conditioned by a spikelet rachis short hair gene on chromosome 7H (Chr7H) [2]; the branched-spike gene *prbs* was mapped to the short arm of chromosome 3H (Chr3H) in the Prbs mutant [7,12]. This gene was flanked by two simple sequence repeat (SSR) markers, Bmag0023 and Cbic60, with genetic distances of 3.3 and 5.4 cM, respectively [7]. In the Xueerdong mutant, the branched-spike gene was flanked by a SSR marker HVM40 and a restriction fragment length polymorphism (RFLP) marker CDO669 with genetic distances of 8.7 and 5.8 cM from the gene on the short arm of Chr4H [3], respectively. The branched-spike gene of the Compositum mutant was mapped to the short arm of Chr2HS and flanked by the M_1_ and M_2_ CAPS markers [10]. Therefore, different genes control the branched spike trait in different barley mutants.

A high-density genetic map is a prerequisite for fine-mapping of barley genes. To date, a large number of molecular markers have been developed based on barley genome sequences, such as RFLP, amplified fragment length polymorphism (AFLP), SSR, sequence-tagged site (STS), single nucleotide polymorphism (SNP) and diversity array technology (DarT) marker. Using these molecular markers, some genetic maps have been successfully used to map the genes or QTLs of barley agronomic traits [13–16], but the accuracy of gene mapping is not sufficiently high. Quickly development of second-generation sequencing technology makes it possible to develop barley SNP markers on a large scale [17]. Using high density SNPs, Jia et al. [18] fine-mapped the gene *ari-e*, which is responsible for barley plant height. Due to the relatively large genome [19] and high sequencing cost [17], the application of whole genome sequencing technology was greatly limited. Therefore, SNP identification and genotyping based on simplified sequencing is a reasonable choice for high-density map construction and gene or QTL fine-mapping in barley. Chutimanitsakun et al. successfully fine-mapped the QTLs of plant height, spike length and grain number per spike using SNPs developed from RAD-seq(restriction-site associated DNA sequence) [20–22]. Zhou et al. [23] developed 12,998 SLAF markers and constructed a high-density genetic map with a total genetic distance of 967.6 cM. Because of their high efficiency, low cost and high resolution [17,24], SLAF markers generated by SLAF-seq have been widely used in the construction of high-density maps and fine-mapping of agronomic trait genes or QTLs in maize [25], cotton [26] and rice [27–28].

The Ynbs line is a novel branched-spike mutant of naked barley generated by ethyl methane sulfonate (EMS) mutation of Beiqing7 and then consecutively selfed for 8 years [6,9]. Compared with other mutants, including Foma, the Ynbs line shows longer and more branched spikes, more single-floret spikelets, and more degenerated spikelets [6]. Wang et al. [9] found that the branched spike of the Ynbs mutant was genetically associated with other traits in the spike, such as the number of single-floret spikelet per spike, seed set rate and number of degenerated spikelet. However, to the best of our knowledge, mapping gene for the branched-spike trait in Ynbs mutant has not been reported to date. In the present paper, the genetic characteristics of the branched spike of the Ynbs mutant were analyzed using F_1_, F_2_ and F_2:3_ materials from the crosses between Ynbs-1 and normal-spike genotypes. A high-density genetic map of barley was constructed based on SLAF markers, and then, the branched-spike gene in the Ynbs mutant was fine-mapped.

## 1. Materials and methods

### 1.1 Experimental materials and phenotype evaluation

Three barley genotypes, Ynbs-1, RIL-1 and BDM-8, were used as parents in this study. Ynbs-1 is a branched-spike mutant from Beiqing7. RIL-1 is a recombination inbred line with six-row spike. BDM-8 is a major six-row-spike barley variety planted in Yunnan province, China, for many years. Three crosses (Ynbs-1/RIL-1, RIL-1/Ynbs-1, and Ynbs-1/BDM-8) were performed to produce F_1_ seeds. The F_2_ populations and their F_2:3_ lines were derived by selfing F_1_ plants of Ynbs/RIL-1 and Ynbs-1/ BDM-8 cross, respectively. All the materials, including the parents, the F_1_ plants (Ynbs/RIL: 56 plants, Ynbs-1/ BDM-8: 82 plants), the F_2_ plants(Ynbs/RIL: 252 plants, Ynbs-1/BDM-8: 635 plants), and the F_3_ lines (Ynbs/RIL: 252 lines, Ynbs-1/ BDM-8: 635 lines), were planted on the experimental farm of Yunnan Agricultural University, with 20 cm per row and 5 cm per plant. The field management was the same as conventional barley cultivation management. At seventh day after flowering, the grains, awns, and glumes were peeled off with tweezers to expose the axis and branched spike, and then, the spike branching characteristics were recorded.

### 1.2 Genomic DNA extraction and marker generation

The young leaves of the two parents (Ynbs-1 and BDM-8) and their 200 F_2_ individuals were collected, and their genomic DNA were extracted by a modified CTAB method [3]. Total DNA was quantified by a spectrophotometer, and the quality of total DNA was evaluated by 1.0% agarose gel electrophoresis with λ DNA as a standard.

SLAF library construction and high-throughput sequencing were conducted according to the method reported by Sun et al. [17]. The barley reference genome (ftp://ftp.ensemblgenomes.org/pub/release-44/plants/fasta/hordeum_vulgare/dna/) was used to design the preliminary restriction enzyme digestion. The restriction enzyme *Hae*III was chosen to digest the genomic DNA of two hybrid parents (Ynbs-1 and BDM-8) and 200 F_2_ individuals. The restriction fragments with a length from 364 bp to 414 bp (with indexes and adaptors) were recovered and purified according to the instruction of a Gel Extraction Kit (50T, Qiagen, Suzhou, China). The gel-purified products were diluted for paired-end sequencing on an Illumina HiSeq 2500 system at Biomarker Technologies Corporation in Beijing, China. Real-time monitoring was performed for each cycle during sequencing, and the ratio of high-quality reads (with quality scores greater than Q30) to raw reads and the G-C content in the raw reads were calculated for quality control. The barcode sequences and terminal 5 bp sequences were trimmed, and then, the clean reads from the same samples were mapped to reference genome sequences using BWA software. All SLAF paired-end reads with clear index information were clustered, and any group of sequences with over 90% identity was considered to be from the same SLAF. Alleles of a SLAF were defined by the minor allele frequency (MAF) evaluation between parents. The MAF value of real genotypes should be extremely significantly higher than that of genotypes containing sequence errors. The SLAF tags showing aa×bb segregation patterns in the F_2_ population were selected, and then, the polymorphic SLAFs with two to four alleles, >10% sequencing depth in the parents and >70% sequence integrity in the offspring were identified as potential markers. The SNP loci of the polymorphic SLAF tags were genotyped with consistency in the parents and offspring.

### 1.3 Map construction and gene mapping

A linkage map was constructed using HighMap software (Develoed by Biomarker Technologies Corporation in Beijing, China) following Liu et al. [22]. The segregation ratio of each marker was calculated by chi-square test, and the markers showing significant (*p* < 0.05) segregation distortion were excluded from map construction. A map region with more than three adjacent loci that showed significant (*p* < 0.05) segregation distortion was defined as a segregation distortion region. The SLAF markers were separated into 7 linkage groups (LGs) corresponding to seven chromosomes in the reference genome. Recombination frequencies and LOD scores were calculated using a three-point test. A combination of enhanced Gibb sampling, spatial sampling and simulated annealing algorithms was used to perform an iterative process of marker ordering. The error correction strategy of SMOOTH was performed according to the parental contribution of genotypes, and the k-nearest neighbor algorithm was applied to impute missing genotypes. The Kosambi mapping function was used to estimate map distances. The genetic map was evaluated by haplotype analysis, linkage assessment and collinearity analysis.

The genome intervals harboring branched-spike genes were identified by the composite interval mapping (CIM) method using the software R/QTL (http://www.rqtl.org/). For the gene locus significantly associated with branched spikes, the genome-wide LOD threshold of 6.344 was determined after 1,000 iteration tests at the 5% significance level.

## 2. Results

### 2.1 Genetic analysis of the branched-spike trait

**Fig 1:**
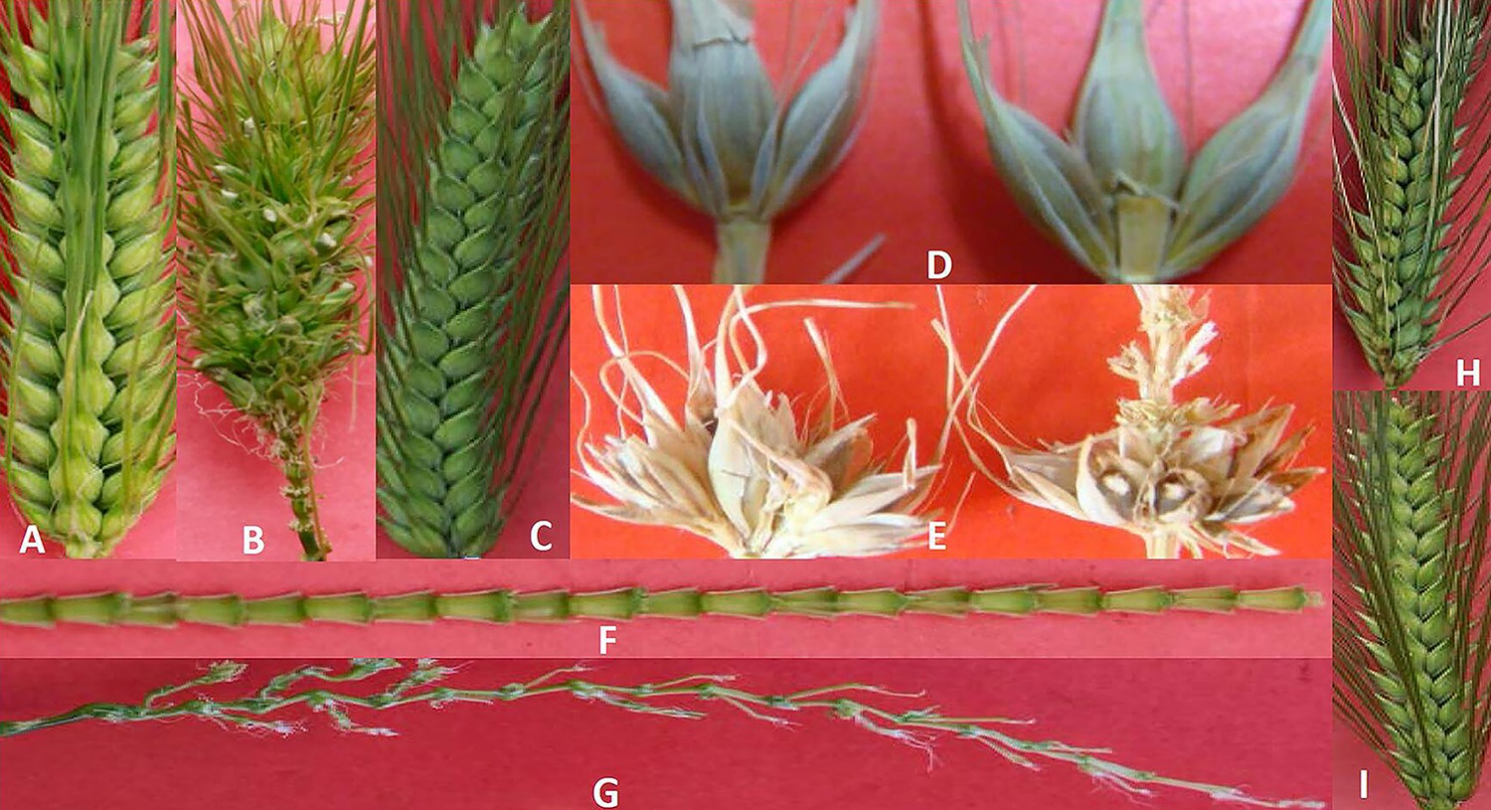
The spike characteristics of BDM-8, Ynbs-1, RIL-1 and F_1_ individual. Fig 1A, B, C, H and I: the spikes of RIL-1, Ynbs-1, BDM-8 and F_1_ individuals from Ynbs-1/RIL-1 and Ynbs-1/BDM-8 cross, respectively; D and E: a six-row spikelet and a multiple-row spikelet, respectively; F and G: a normal rachis and a branched-spike rachis, respectively.

The spike of Ynbs-1 had a different structure from those of RIL-1 and BDM-8. Spikes of Ynbs-1 were branched on the spike rachis nodes (Fig 1B and 1G), but spikes of RIL-1 and BDM-8 were not (Fig 1A, 1C and 1F). The rachis of the branched spike in the middle of the main spike was longer than that at the end of the main spike in Ynbs-1 (Fig 1G). Triple spikelet grew on the main spike rachis in RIL-1 and BDM-8 (Fig 1A, 1C, 1D and 1F), but multiple spikelet grew on the branched-spike rachis of Ynbs-1 (Fig 1B, 1E and 1G). The F_1_ plants from all the crosses showed six-row spike (Fig 1H and 1I), indicating that the branched-spike gene of Ynbs-1 is a recessive nuclear gene. The individuals in the F_2_ population from the crosses Ynbs-1/RIL-1 and Ynbs-1/BDM-8 segregated with 1:3 branched-spike to six-row-spike plants at a 1% significance level (Table 1), indicate that the branched-spike trait of Ynbs-1 is genetically controlled by a recessive nuclear gene. To verify this result, all the F_2_ individuals were bagged and self-pollinated to create F_3_ lines. The χ^2^ test of segregation in F_3_ lines supported single recessive gene hypothesis from the F_2_ population (Table 1).

**Table 1:** Spike type segregation in the F_2_ and F_3_ generations of crosses Ynbs/RIL-1 and Ynbs/BDM-8.

Cross	Number of F_2_ plant with normal spike		Number of F_2_ plant with branched spike	χ^2^ value	
	Non segregating line in F_3_ generation	Segregating line in F_3_ generation	Non segregating line in F_3_ generation	1:3	1:2:1
Ynbs/RIL-1	60	130	62	0.0053	0.29
Ynbs/BDM-8	160	329	146	1.26	1.45
Total	220	459	208	1.06	1.41

### 2.2 Generation of SLAFs markers

A total of 51.63 Gb of raw reads were obtained by next-generation sequencing, which generated 2,048,713,462 paired-end reads at 200 bp in length. The G-C content was 47.81%, and the Q_30_ reached 93.08% in the raw data. A total of 35,936,800 reads generated 668,042 unique SLAFs for Ynbs-1, with the average coverage of 26.85-fold for each SLAF. Similarly, the numbers of reads and SLAFs from BDM-8 were 37,383,576 and 663,784, respectively, with the average coverage 27.74-fold for each SLAF. In the F_2_ population, 9,876,965 reads generated 434,681 SLAFs per progeny with an average coverage of 11.65 (Table 2).

**Table 2:** Characteristics of SLAF-seq data for the three materials.

Sample	Total read	SLAF number	Total depth	Average depth
BDM-8	35,936,800	668,042	17,933,629	26.85
Ynbs-1	37,383,576	663,784	18,412,657	27.74
Offspring mean	9,876,965	434,681	5,062,042	11.65

**Fig 2:**
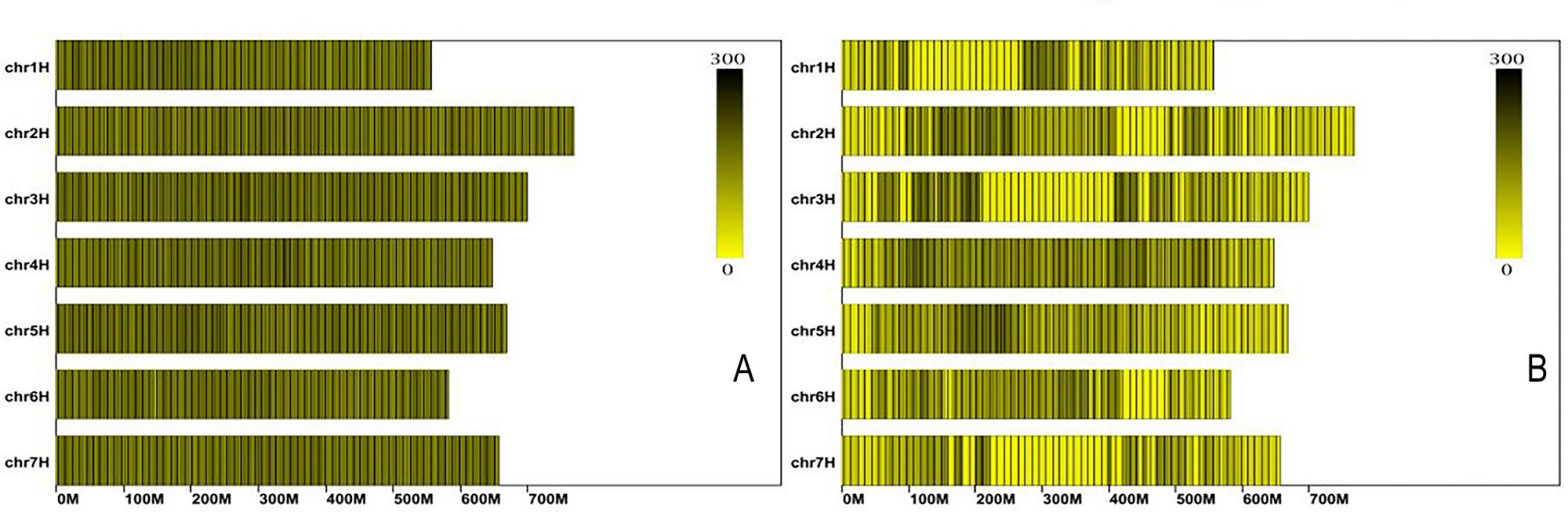
Distribution of SLAF tags on the barley genome. The x-axis: chromosome length, and the y-axis: chromosome code. The trend from yellow to black: SLAF number change from less to more. Fig 2A:the distribution of all 885,499 high-quality SLAFs. Fig 2B: the distribution of 210,077 polymorphic SLAFs.

After filtering out low-quality and low-depth SLAFs, a total of 753,189 high-quality SLAFs were obtained. Of these, 227,624 SLAF tags were polymorphic with the polymorphism rate of 30.22% (Table 3). The polymorphic SLAFs were allocated to the seven chromosomes of barley, with each chromosome having 22,398 to 43,417 polymorphic SLAFs (Table 3, Fig 2). SLAFs with polymorphisms were genotyped for both parents and F_2_ individuals. A total of 135,576 SLAFs showed aa×bb segregation among the F_2_ individuals. SLAFs with parental sequencing depth less than 14-fold, integrity less than 99%, and significant segregation distortion (*p* < 0.05) were filtered out, and 14,348 SLAF markers were finally selected for linkage map construction (Table 4).

**Table 3:** Distribution of SLAF tags on the 7 barley chromosomes.

Chr ID	SLAF number	Polymorphic SLAFs	Percentage of polymorphic SLAFs (%)
Chr1h	94,607	22,398	23.67
Chr2h	120,874	36,955	30.57
Chr3h	113,352	31,403	27.70
Chr4h	118,915	43,417	36.51
Chr5h	99,782	36,891	36.97
Chr6h	96,064	29,943	31.17
Chr7h	101,322	24,469	24.15
Other	8,273	2,148	25.96
Total	753,189	227,624	30.22

**Table 4:** Genetic characteristics of the 7 barley linkage groups (LGs).

LGs	No. of SLAFs	No. of SNPs	Total distance (cM)	Average distance between SLAFs (cM)	Largest gap (cM)
Chr1H	1,144	1,949	165.27	0.14	6.23
Chr2H	2,049	3,443	207.23	0.10	4.69
Chr3H	5,445	8,753	213.10	0.040	3.07
Chr4H	1,154	1,995	192.72	0.17	4.43
Chr5H	1,970	3,437	200.91	0.10	4.28
Chr6H	1,292	2,147	196.91	0.15	3.53
Chr7H	1,294	2,183	171.30	0.13	3.37
Total	14,348	23,907	1,347.44	0.090	6.23

### 2.3 Construction of a genetic map

**Fig 3:**
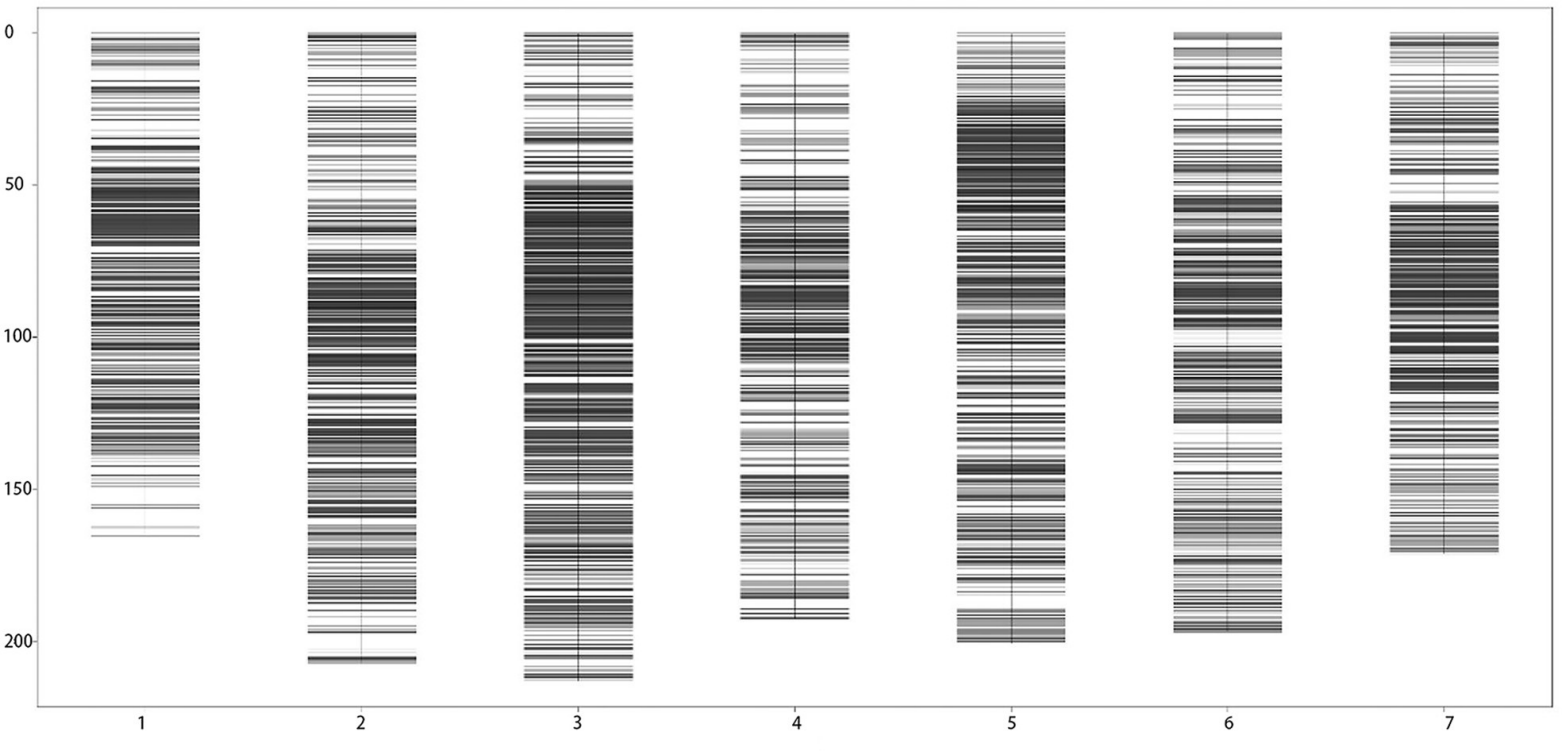
Distribution of SLAF markers on 7 barley linkage groups. Black bars: SLAF markers, x-axis: linkage group code, and y-axis: map distance (cM).

After the linkage analysis, a genetic map with a total length of 1,347.44 cM in 7 linkage groups and an average distance of 0.090 cM between adjacent markers was finally obtained. The genetic lengths of the 7 linkage groups ranged from 165.27 cM (Chr1H) to 213.10 cM (Chr3H), with an average distance between adjacent markers from 0.04 cM to 0.17 cM per chromosme. Chr3H was the most saturated, containing 5,445 SLAF markers and covering a length of 213.10 cM, with a 0.04 cM average distance between adjacent markers. In contrast, Chr4H was the least saturated, with 1,154 SLAF markers and a 0.17 cM average distance between adjacent markers. The maximum gap was 6.29 cM between Marker14110890 and Marker14104365 on Chr1H. Detailed information of the genetic map is presented in Table 4 and Fig 3.

A haplotype map of the 14,348 markers was generated to detect double crossovers and the genotyping errors and recombination events (S1 Fig). Most of the recombination blocks were clearly defined with a few double recombinations or deletions in the 7 linkage groups. The linkage between adjacent markers was very strong in each linkage group (S2 Fig). The linkage between markers was gradually weakened as increase in genetic distance. These results suggested that most linkage groups did not undergo frequent recombination (Table 5).

**Table 5:** The percentage of double crossover or deletion SLAF markers of 7 barley linkage groups.

LG ID	Singleton (%)	Miss (%)
Chr1h	0.040	0.12
Chr2h	0.0010	0.0012
Chr3h	0.0010	0.0011
Chr4h	0.010	0.20
Chr5h	0.010	0.010
Chr6h	0.020	0.090
Chr7h	0.010	0.020

**Fig 4:**
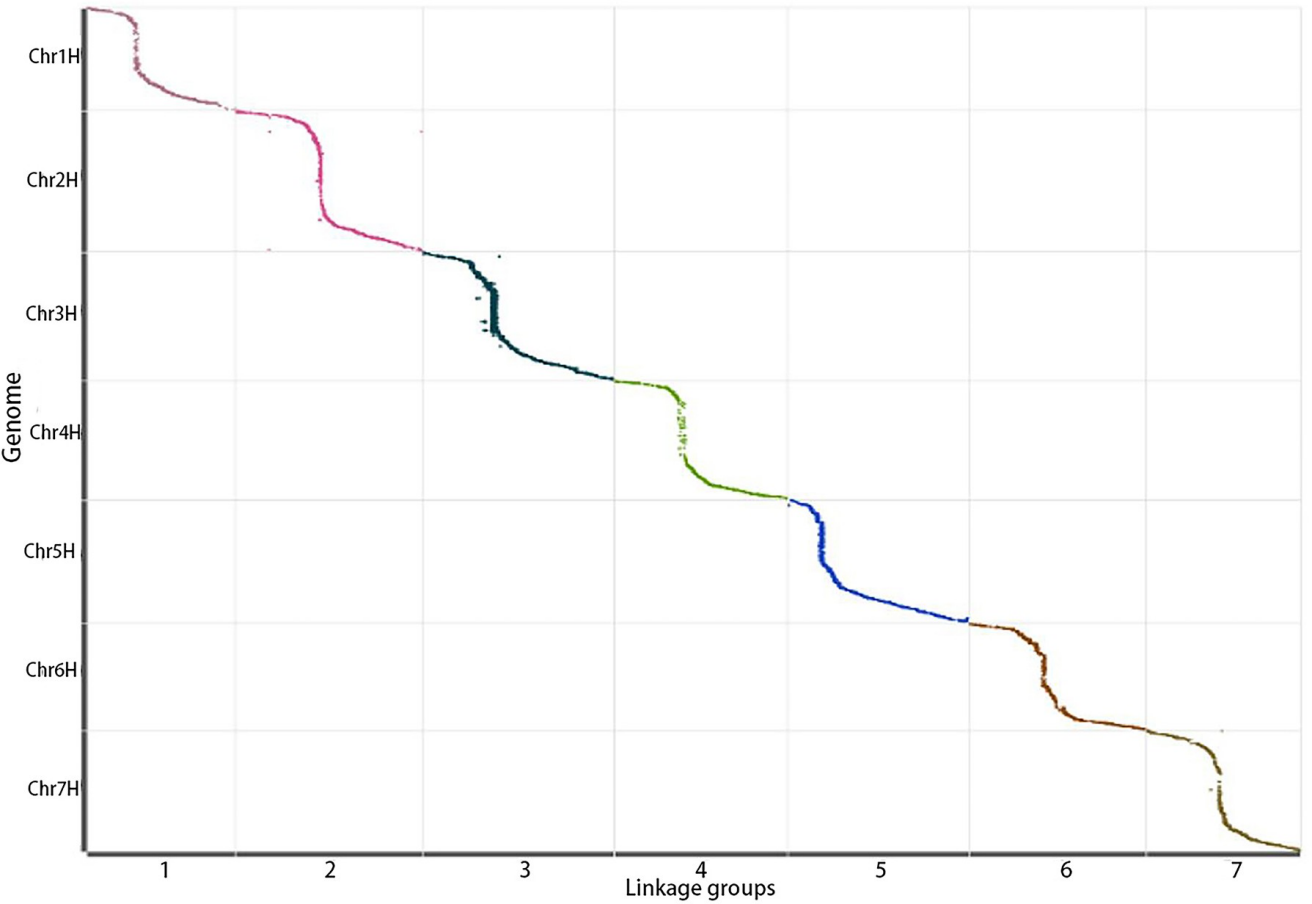
The collinearity between 7 linkage groups and their physical maps. X-axis: the genetic distance of each linkage group, y-axis: the physical position in the barley genome. Different colors represent different linkage groups. The collinearity of marker in genome and genetic map was expressed in the form of scattered points.

All the mapped SLAF markers were anchored to the barley reference genome to evaluate the collinearity of the genetic linkage with the reference genome. High collinearity between the linkage groups and their physical maps was observed, the coefficients of collinearity ranged from 0.9925 to 0.9998 (*p* < 0.05), with a falling trend in most parts of the collinear curves (Fig 4), indicating identical marker order between their genetic linkage and physical maps. The continuity of the collinear curves generated from the 7 linkage groups indicated that the genome was sufficiently covered by the SLAF markers, and the SLAF markers were mapped accurately within each linkage group.

### 2.4 Map gene for the branched-spike trait

**Fig 5:**
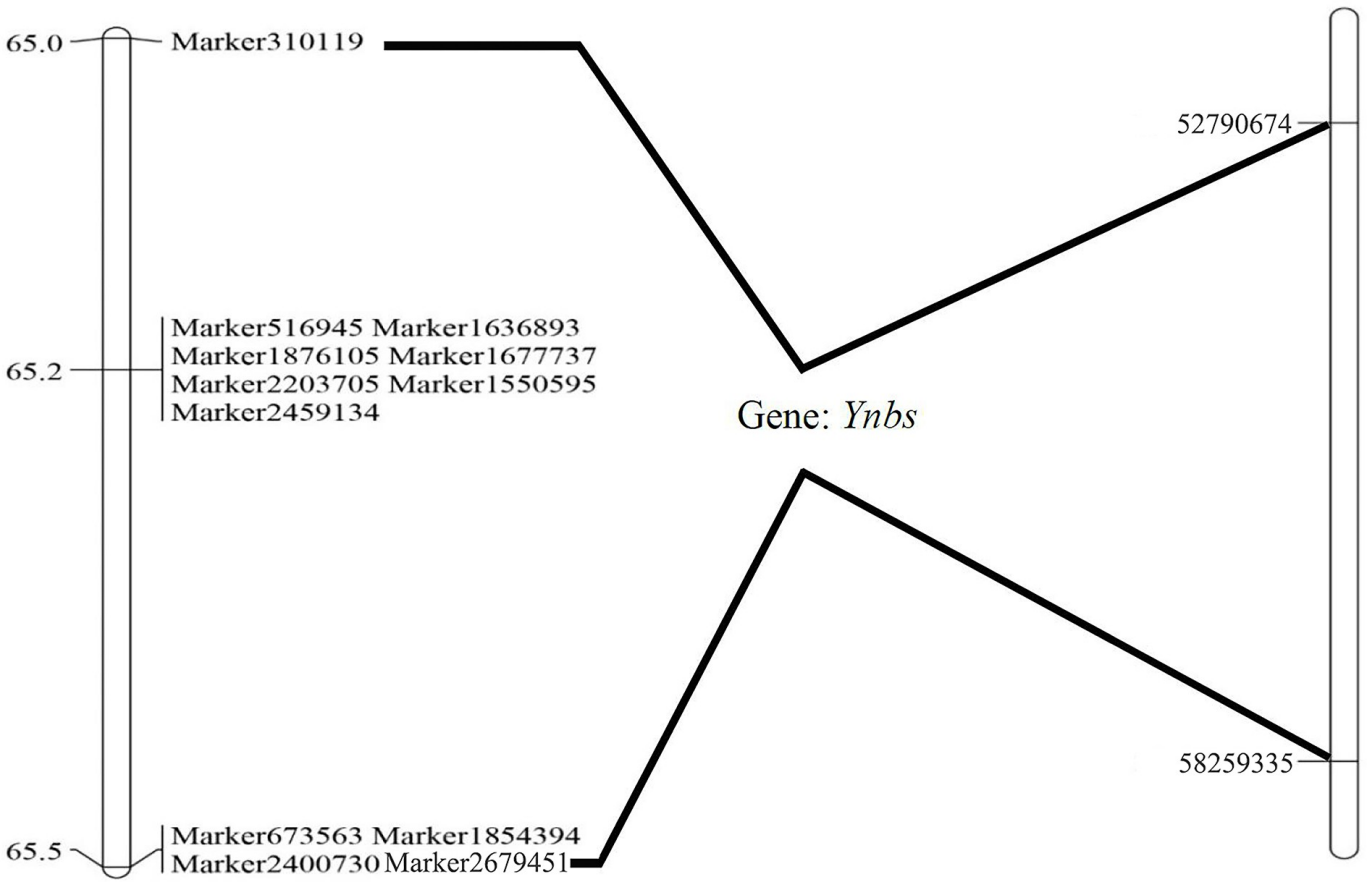
A gene for the branched-spike trait in the Ynbs mutant mapped on barley Chr2H. Genetic (left) and physical (right) location of *ynbs* gene.

Only one branched-spike gene locus with an LOD value of 67.53 was found; it was located on Chr2H of barley and could explain 74.66% of the phenotypic variation. The locus was flanked by Marker310119 and Marker2679451, ranging from 65.00 cM to 65.47 cM. Twelve SLAF markers were mapped in the candidate interval, with 1, 7, and 4 SLAF markers mapped at 65.00, 65.22 and 65.47 cM, respectively. The alignment analysis of the 12 SLAF markers to the barley reference genome suggested that the gene locus was located in a 5.47 Mb physical interval between 52,790,674 bp and 58,259,335 bp (S1 Table) on Chr2H (Fig 5).

## 3. Discussion

At seventh day after flowering, we found that the branched spikes on different spike rachises of Ynbs-1 showed different lengths. The branched-spike length, branched spike number and branched-spike rachis node number of Ynbs-1 were 1.48±0.86cm, 13.56± 1.52 and 2.32±0.63, respectively (S2 Table) [6,9]. Ji et al. found that the branched spikes of the mutants Prbs [4], F_151_[3]and Compositum [10] grew on 1^st^ and 2^nd^ spike rachis nodes on the main spike, with 1–2 nodes of branch-spike rachis. Compared with these other mutants, Ynbs-1 had more and longer branched spikes.

The genetic analysis of the F_1_ plants from the reciprocal crosses between branched-spike mutants and six-row-spike materials indicated that the branched-spike trait in this study was controlled by a nucler gene and not affected by cytoplasmic genes,. Further analysis of F_2_ populations and their F_3_ lines suggested that the branched spike trait in Ynbs-1 was controlled by single recessive gene, which confirmed previous reports for mutants F_151_ and Prbs [3,11].

Based on 14,348 SLAF markers, a high-density genetic map of barley was constructed with a total length of 1,347.44 cM. Many high-density genetic maps have been constructed using SSR, DarT and SNP markers for different barley populations [21–22,29–31]. Of them, the high-density genetic map constructed by Wang et al. [21] had relatively high quality, with 1,375.8 cM length and a 0.7 cM mean adjacent marker distance. Compared with other genetic maps, the map reported in the present paper had significantly improved marker density and marker uniformity.

Gene mapping using the linkage map only detected one gene on Chr2H for branched-spike. This result was consistent with the phenotypic segregation ratio derived from the F_2_ population and F_3_ lines of Ynbs-1/RIL-1 and Ynbs-1/BDM-8. Poursarebani et al. [10] also found a branched-spike gene on Chr2HS, in which the *com2* gene was responsible for the branched spike of the Compositum mutant. Sequence alignment of specific primer sequences to barley reference genome showed that *com2* was located in a genome region from 54,784,028 to 54,793,302 bp. However, the phenotype of the Ynbs mutant is different from that of the Compositum mutant [6,10]. Therefore, we speculate that the heredity of spike branching in the Ynbs mutant may be controlled by the gene other than *com2*.

Using reference genome annotation, we found 341 genes which include *F-box gene*, *MYB gene* and *receptor protein kinase gene*, in the branched-spike candidate gene region of the Ynbs mutant (S3 Table). To date, many reports have indicated that *F-box* genes, such as the rice *APO1* and *UFO* genes (unusual floral organs), are involved in regulating flower organ morphology, transformation from inflorescence meristem to spikelet meristem [32], inflorescence branching or floral organ meristem identification [33]. In rice, the leucine receptor protein kinase gene *LRK2* also participated in the regulation of rice branching, and its ectopic expression could effectively increase the tiller number of rice [34]. Boisson-Dernier et al. [35] found that the receptor protein kinase genes *ANX1* and *ANX2* were essential for the growth of the pollen tube and root tip apex in *Arabidopsis thaliana*, and the mutation of these genes could lead to the rupture of the pollen tube and a decreased seed set rate. DeYoung et al. [36] verified that the receptor protein kinase genes *BAM1*, *BAM2* and *BAM3* were related to the *Arabidopsis thaliana* meristem. Mutations in *BAM1*, *BAM2* and *BAM3* could cause phenotypic aberrations, such as abnormal male gamete development and leaf loss, which were similar to those produced by the loss of stem cells in stem and flower meristem tissues. Liu et al. [37] found that mutation of the class III lipase gene *This1* could effectively increase tiller, reduce plant height and floret fertility in rice. Because of the defect in pollen maturation, anther dehiscence and flowering, the seed set rate of the rice *This1* mutant was less than half that of a wild-type plant. Therefore, we speculate that the branched-spike gene locus of the Ynbs mutant harbors some genes regulating the heredity of branch-spike, spikelet, floret and seed setting rate traits, respectively.

A significant association of branched spikes with multiple-spikelet degeneration, single-floret spikelet increase, and seed set rate decrease has been demonstrated in the Ynbs mutant [6,9]. In the present study, we found that the genetic distance and physical distance between flanking markers for the branched-spike gene of the Ynbs mutant was only 0.47 cM and 5.47 Mb (Chr2H: 52790674–58259335), respectively. The branched-spike gene region was divided into two parts at 65.20 cM, with 7 SLAF markers clustered together, and recombination between these markers was not efficiently detected by above linkage analysis, and the region harbors some genes, which controll the heredity of branched spike, triple- or mult-spikelet number, floret number and seed setting rate. Therefore, the branched-spike genes of the Ynbs mutant may be highly linked to the genes controlling spikelet, floret, and seed set rate, and this linkage may cause the genetic association of the branched-spike trait with the above mentioned traits.

## Supporting information

S1 Fig Recombination events in 200 F_2_ offspring and parental lines. Each row represents a marker arranged in the order of position on the linkage group from the short arm (top) to the long arm (bottom) of the chromosome, and each chromosome of each individual is shown in the column. Green, blue and red indicates female parent, male parent and heterozygosity, respectively. The color change in the same column represents a recombination event.
(TIF)

S2 Fig Recombination frequency between markers in each linkage group. From top-left to the bottom-right, each row and column is a marker arranged in the order of linkage group. Each cell represents the recombination rate between markers. Yellow, red and purple indicates the minimum, median and maximum recombination rate, respectively.
(TIF)

S1 Table Physical and genetic locations of the candidate gene interval. (XLS)

S2 Table Branched-spike length, branched spike number and branched-spike rachis node number of Ynbs-1. (XLS)

S3 Table Integrated functional annotation of the putative genes in the candidate gene region. (XLS)
