# Supplementary figures and images for: Construction of a high-density genetic map and fine mapping of a candidate gene locus for a novel branched-spike mutant in barley
Source: PLoS One. 2020 Jan 8;15(1):e0227617. doi: 10.1371/journal.pone.0227617 (PMC6948822; doi:10.1371/journal.pone.0227617)

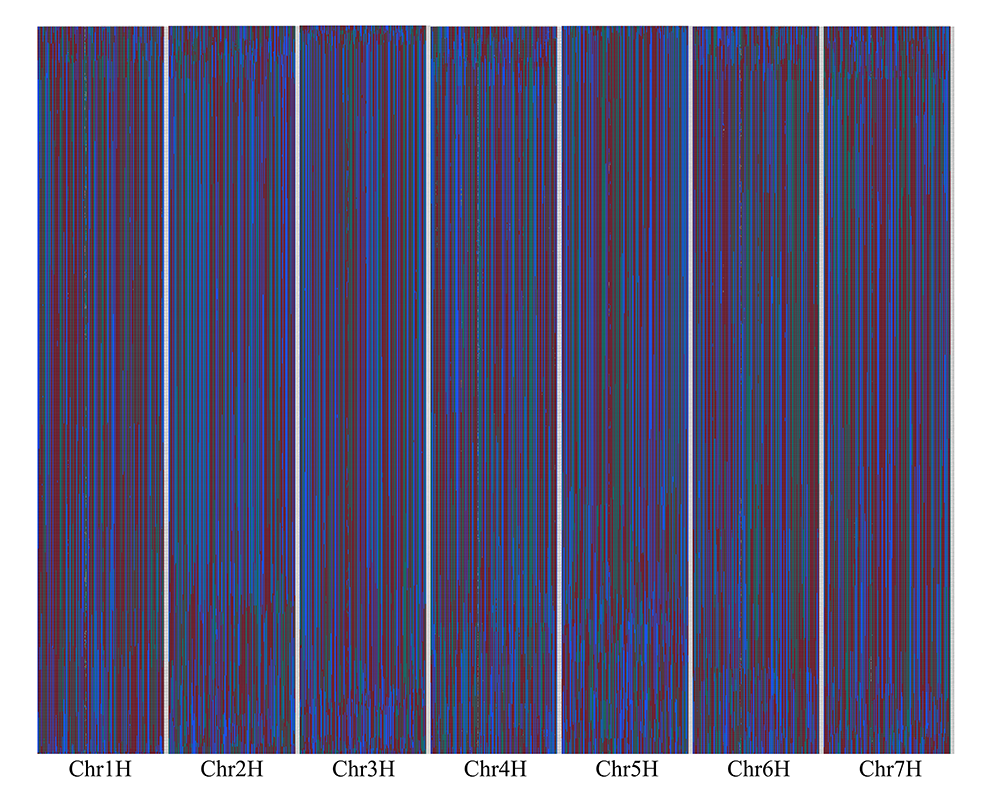

Supplement: S1 Fig — Each row represents a marker arranged in the order of position on the linkage group from the short arm (top) to the long arm (bottom) of the chromosome, and each chromosome of each individual is shown in the column. Green, blue and red indicates female parent, male parent and heterozygosity, respectively. The color change in the same column represents a recombination event. (TIF) [file pone.0227617.s001.tif]

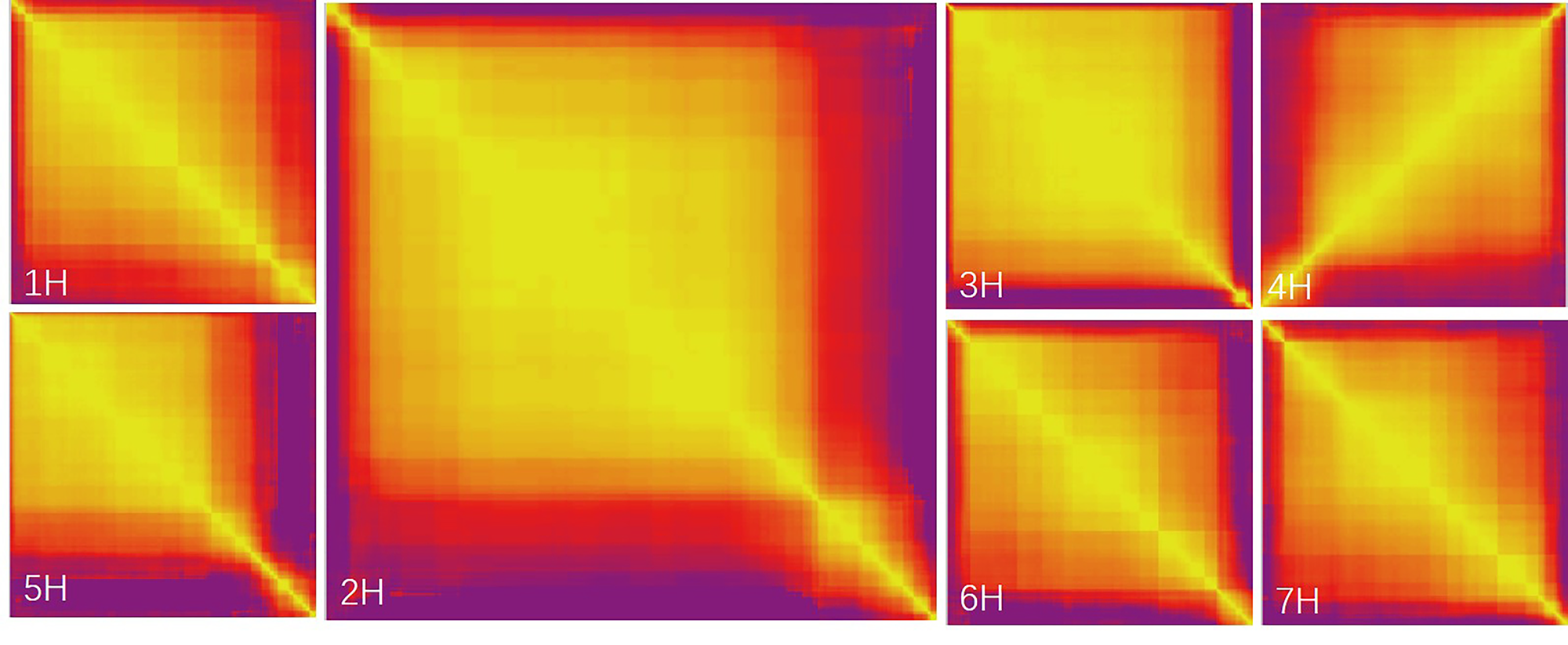

Supplement: S2 Fig — From top-left to the bottom-right, each row and column is a marker arranged in the order of linkage group. Each cell represents the recombination rate between markers. Yellow, red and purple indicates the minimum, median and maximum recombination rate, respectively. (TIF) [file pone.0227617.s002.tif]
